# Fluorescence Analysis of Vitamin D Receptor Status of Circulating Tumor Cells (CTCS) in Breast Cancer: From Cell Models to Metastatic Patients

**DOI:** 10.3390/ijms18061318

**Published:** 2017-06-20

**Authors:** Xi Zhang, Simone Hofmann, Brigitte Rack, Nadia Harbeck, Udo Jeschke, Sophie Sixou

**Affiliations:** 1 Department of Obstetrics and Gynaecology, Breast Center, Ludwig-Maximilians University of Munich (LMU), Maistrasse 11, 80337 Munich, Germany; Xi.Zhang@med.uni-muenchen.de (X.Z.); Simone.Hofmann@med.uni-muenchen.de (S.H.); brigitte.rack@uniklinik-ulm.de (B.R.); Nadia.Harbeck@med.uni-muenchen.de (N.H.); Udo.Jeschke@med.uni-muenchen.de (U.J.); 2 Faculty of Pharmacy, University Paul Sabatier Toulouse III, Toulouse CEDEX 09 31062, France

**Keywords:** vitamin D receptor, circulating tumor cells, breast cancer

## Abstract

The Vitamin D receptor (VDR) expressed in normal breast tissue and breast tumors has been suggested as a new prognostic biomarker in breast cancer (BC). Besides, increasing evidence supports the view that the detection of circulating tumor cells (CTCs) predicts outcome in early and metastatic BC. Consequently, an evaluation of VDR expression in the CTCs of BC patients may allow optimization of their treatment. As an attempt to profile and subtype the CTCs of metastatic patients, we established an innovative fluorescence technique using nine BC cell lines to visualize, define, and compare their individual VDR status. Afterwards, we tested the CTC presence and VDR expression in blood samples (cytospins) collected from 23 metastatic BC patients. The results demonstrated major differences in the VDR levels among the nine cell lines, and VDR positive CTCs were detected in 46% of CTC-positive patients, with a total of 42 CTCs individually analyzed. Due to the limited number of patients in this study, no correlation between VDR expression and BC subtype classification (according to estrogen receptor (ER), progesterone receptor (PR) and HER2) could be determined, but our data support the view that VDR evaluation is a potential new prognostic biomarker to help in the optimization of therapy management for BC patients.

## 1. Introduction

Breast cancer (BC) is a significant global public health issue and the leading cause of death among women around the world. Anti-cancer therapies including chemotherapy, endocrine therapy, and targeted therapy have significantly decreased BC mortality in the past 20 years. However, as BC is an extremely heterogeneous disease, resistance to treatment is a major clinical challenge for current BC management [1]. Therefore, the development of more specific biomarkers and recognition of new therapeutic targets would really contribute to solving the problems of therapy resistance and metastasis [2,3,4]. In recent years, an increasing number of clinical studies have suggested that an optimal vitamin D status has a protective effect against BC development and that high Vitamin D receptor (VDR) expression in breast tumors is associated with a better survival rate [5,6,7,8]. As one of the nuclear receptor (NR) members, VDR is found in both normal breast tissue and breast tumors [9,10]. As such, an analysis and understanding of the VDR pathway can probably provide a novel way for developing a new targeted therapy to escape resistance mechanisms. The group of NRs that are active as homodimers have been classified as type 1 NRs, whereas the NRs of the VDR group that bind as heterodimers are known as type 2 NRs. The type 1 group includes, among others, estrogen, progesterone, and androgen receptors and the type 2 group contains VDR, retinoic acid receptors (RARs), retinoid X receptors (RXRs), and thyroid hormone receptors (THRs). Ligand-bound VDR-activated vitamin D_3_ heterodimerizes with its cognate co-receptor RXR to control the expression of genes involved in its different functions [11]. Besides its classic functions to maintain extracellular calcium levels by regulating calcium absorption in the gut and bone turnover, the VDR–RXR heterodimer binds to vitamin D response elements with the positive or negative transcriptional regulation of gene expression involved in various molecular pathways. This results in a wide range of calcitriol-mediated anti-cancer actions in BC [12,13]. We therefore believe that VDR exploration is very relevant to evaluate its potential as a new prognostic biomarker and therapeutic target in BC.

Circulating tumor cells (CTCs) circulate in the peripheral blood of patients with solid malignancies and are shed from an existing primary tumor or from metastatic lesions into the blood stream [14]. CTCs detected in BC patients are significantly associated with a poor outcome in both early and metastatic tumors [15,16,17,18,19]. In metastatic patients, several tumor lesions may potentially release CTCs which therefore comprehensively reflect tumor and metastasis characteristics. CTCs can be collected via a simple venipuncture; this ‘liquid biopsy’ achieves the repeatable and real-time monitoring of tumor cell characteristics. It is a less invasive and cost-effective alternative to tissue biopsies [20], despite the fact that technical and conceptual advances are still necessary before this ‘liquid biopsy’ can be routinely used for the diagnosis, characterization, monitoring, and treatment optimization of cancer. CTCs are a promising marker, providing important predictive and prognostic information in both early and metastatic BC. They may help to assess the response to treatment and to detect early disease recurrence [21]. At the moment, the CellSearch^®^ system for CTC enumeration is the only accepted standard by the Food and Drug Administration (FDA). Only a few studies have investigated human epidermal growth factor receptor 2 (HER2) and/or estrogen receptor (ER) expression on CTCs, even though HER2 and ER are currently the only validated predictive factors used for therapy decision making in BC [22]. In conclusion, the characterization of CTCs may be a major tool to support diagnosis, and should be included in clinical trials for the evaluation of new targeted therapies [23]. In order to better predict disease progression and personalize treatment, new prognostic and predictive factors are needed. So far, studies on VDR status in CTCs are still lacking. Therefore, the evaluation of VDR expression on CTCs in BC patients could potentially help in individualizing BC therapy.

In this study, we describe an innovative triple fluorescence technique that we developed to simultaneously visualize the presence of cytokeratin (CK), absence of CD45, and expression of VDR. We first characterized BC cell models, before validating the preclinical data in CTCs from 23 metastatic BC patients.

## 2. Results

### 2.1. Fluorescence Labeling of VDR, CK, and CD45 on MCF-7 Cells Mixed with Peripheral Blood Mononuclear Cells (PBMCs)

**Figure 1:**
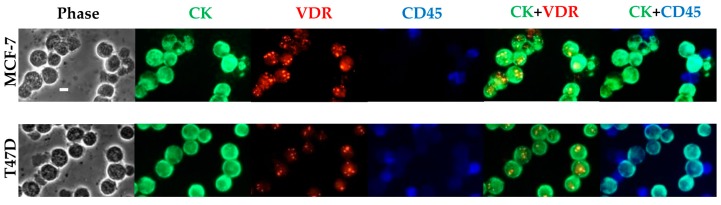
VDR expression detection in MCF-7 and T47D cells mixed with PBMCs. Fluorescence labeling of VDR (in red), CK (in green), and CD45 (in blue) was performed on a 10^6^ MCF-7 and T47D cells/PBMCs mix (3/1). Original magnification, ×40. Scale bar (white bar in the upper left image), 10 μm. CK: cytokeratin; VDR: vitamin D receptor; PBMCs: peripheral blood mononuclear cells.

To mimic the physiological situation of identifying very few CTCs within numerous PBMCs, we first used MCF-7 and T47D cells as models for VDR-positive BC cells and mixed them with PBMCs from healthy donors (Figure 1). A 40× magnification allowed us to simultaneously visualize, in both cell lines, the ring-like appearance of CK in green and the nuclear staining of VDR in red labeling, as well as the blue ring-like appearance of CD45 in the PBMCs. The optimized staining protocol allowed us to screen for cancer cells expressing CK and no CD45 and distinguish them from the CD45 positive PBMCs. VDR expression could then be assessed in cancer cell lines.

### 2.2. Fluorescence Labeling of VDR and CK with Parallel 4′-6-Diamidino-2-Phenylindole (DAPI) Staining in MCF-7 Cells and in a Panel of Eight other BC Cell Lines and One Endometrial Cancer Cell Line

**Figure 2:**
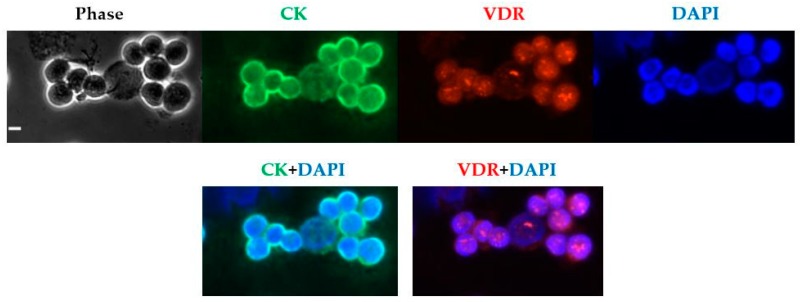
VDR expression detection in MCF-7 cells. Fluorescence labeling of VDR (in red) and CK (in green) with DAPI (in blue) nuclear staining was performed on 10^6^ MCF-7 cells. Original magnification, ×40. Scale bar (white bar in the upper left image), 10 µm. DAPI: 4′-6-Diamidino-2-Phenylindole.

To confirm VDR nuclear staining, the fluorescence labeling of VDR and CK with DAPI nuclear staining was then performed on MCF-7 cells (Figure 2). The previously described aspect of first nuclear foci of VDR was characterized [24,25]. It is noteworthy that not all MCF-7 cells exhibited the same intensity of VDR staining. Whereas some cells barely exhibited any fluorescence intensity (roughly 3%), most exhibited significant fluorescence and others expressed a particularly high intensity (around 4%).

**Figure 3:**
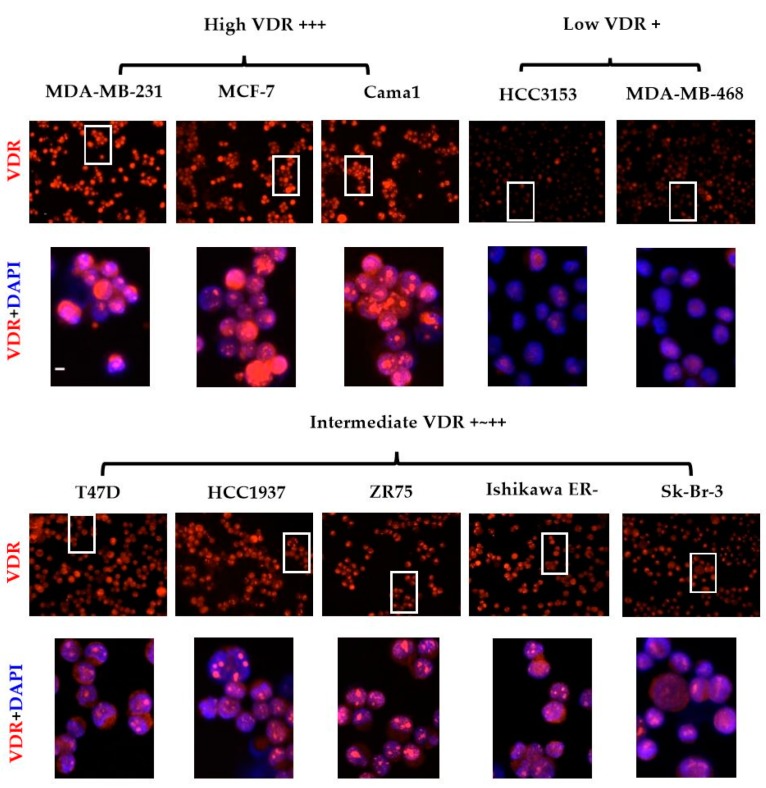
VDR expression in nine BC and one endometrial cell lines. Fluorescence labeling of VDR (in red), CK with DAPI (in blue) nuclear staining was performed on 10^6^ cells from 10 cell lines. Various levels of VDR expression were observed, including low (+), average (+~++), or high (+++) levels. Photographs presented are representative of five independent reproducible experiments. Original magnification, ×40. Scale bar (white bar in the upper left image), 10 μm. BC: breast cancer.

As presented in Figure 3, we then performed the labeling using this protocol on nine different human BC cell lines (MCF-7, T47D, Cama-1, ZR75, SK-Br-3, HCC 3153, HCC1937, MDA-MB-231, and MDA-MB-468) and one endometrial cancer cell line Ishikawa ERneg, to analyze the differences in VDR expression. For each cell with systematic CK labeling, we were able to characterize different VDR levels by evaluating the average intensity: low VDR: +, intermediate VDR: +~++ and high VDR: +++. Because VDR expression could exhibit clear differences within one cell line, like MCF-7, we assessed the average intensity observed for the majority of cells within each cell line. According to our results, all 10 cell lines then appeared as VDR positive. However, as shown in Table 1, we observed that the different cell lines expressed very different average levels of VDR. The following grading was obtained: high VDR +++ includes MDA-MB-231, MCF-7 and Cama-1; intermediate VDR +~++ includes T47D, HCC1937, ZR75 and SK-Br-3; low VDR + includes MDA-MB-468 and HCC 3153. In addition, Ishikawa ERneg belonged to intermediate VDR +~++.

### 2.3. Patient Cohort

A cohort of 23 metastatic BC patients was included in the study. The clinicopathological data are shown in Table 2, with an average age of 64.9 years at the time of CTC analysis (median: 66 years; range: 46–82 years). Patients M10 and M25 had bilateral BC: While patient M10 had two ERα- and HER2 positive tumors, patient M25 had an ERα positive and HER2-negative tumor in the left breast and one ERα- and HER2-negative tumor in the right breast. Their tumors were considered as two independent primary tumors for statistical analysis. In total, 68.0% of the primary tumors were ERα positive (*n* = 17), 36.0% were HER2 positive (*n* = 9, with four patients both ERα and HER2 positive), and 12.0% were triple-negative (*n* = 3). At least 76.0% of the tumors were grade 2 or 3 at the time of primary diagnosis (*n* = 19). The first metastasis was diagnosed at an average of 3.5 years after primary diagnosis (median: 3 years; range: 0–10 years). CTC analysis was performed at an average of 9.8 years after primary diagnosis (median: 10 years; range: 4–16 years) and 6.3 years after the first metastasis (median: 5 year; range: 4–15 years).

### 2.4. CTCs Screening and Enumeration

We found CTCs in 14 patients out of our 23 metastatic BC patients (60.8%). Patient M1 exhibited numerous CTCs which we estimated as more than 500 in the 1 million PBMCs that we analyzed on one cytospin. In addition, 42 CTCs were identified in the other 13 patients, with numbers ranging from one to 16 per patient and an average of 3.2 CTCs per patient (Table 3). Five patients had more than one CTC, with only one patient having more than 10 CTCs.

### 2.5. VDR Status Determination in CTCs

**Figure 4:**
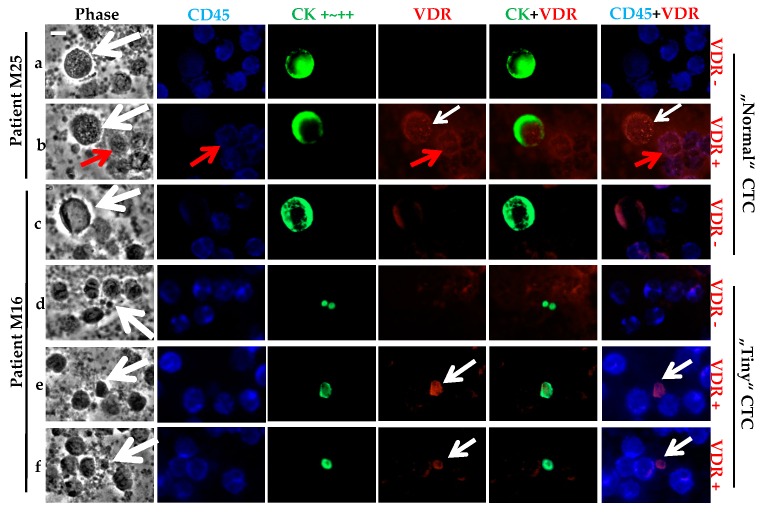
VDR status determination on CTCs of metastatic BC patients. Triple fluorescence labeling of CD45 (in blue), CK (in green), and VDR (in red) was performed on 10^6^ PBMCs, with parallel phase analysis. CTCs (**with white arrows**) were classified as VDR+ or VDR-. For both patients M25 (**a**,**b**) or M16 (**c**–**f**), either status was observed with superimposed VDR and CK labeling. CTCs exhibit size heterogeneity for patient M16 (“Normal” or “Tiny” CTCs). VDR staining was also seen on PBMCs (**with red arrows**), with superimposed VDR and CD45 labeling. Original magnification, ×40. Scale bar (white bar in the upper left image), 10 μm.

As observed in the cancer cell line models, the strong CK staining allowed the screening of the CD45 negative CTCs (Figure 4). VDR staining was very high in some cases. Based on the cancer cell line controls, we classified two VDR staining statuses for the CTCs: positive if low, moderate, or high expression; or negative. The panels a and b in Figure 4 show the presence of both VDR positive and negative CTCs for the same patient, M25. Besides some VDR positive CTCs, we can see some CD45 positive cells that also expressed VDR (panel b). Similarly, for patient M16, both VDR positive and negative CTCs were seen (panels e and f versus c and d). For the same patient, M16, clear differences in the size of the CTCs occurred, with what we classified as tiny “CTCs” (panels d, e and f) of around a 5 μm diameter, compared to the so-called “normal” CTCs (panels c, around a 10–15 μm diameter).

For patient M1 (Table 3), no accurate quantification of the CTC number was possible, as more than 500 CTCs were identified within the 1 million PBMCs analyzed. This specific subtype of CTCs exhibited a regular size (around 10 μm) with positive or negative VDR expression. Of the remaining 13 patients with CTCs (Table 3), five had only one CTC that was VDR negative, and two patients had two or five CTCs that were all VDR negative. Altogether, seven patients out of 13 (53.8%) only had VDR negative CTCs, three patients (23.1%) had only one CTC that was VDR positive, and the last three patients (23.1%) had both VDR positive and negative CTCs.

Of the total 42 CTCs analyzed, 54.8% (*n* = 23) CTCs were classified as VDR negative and 45.2% (*n* = 19) as VDR positive.

We noticed that almost all patients exhibited round shaped CTCs, as expected after the cytospin preparation of the blood samples. Regarding the average size of the CTCs, eight patients had what we defined as “normal” CTCs (*n* = 18) with diameters ≥5 μm (as described above for panels a to c in Figure 4), whereas nine patients had tiny CTCs (*n* = 24) having a diameter <5 μm (panels d to f in Figure 4). The four patients with more than two CTCs had both tiny and normal size CTCs. Both populations of tiny and normal CTCs could equally express VDR or not express VDR. We noticed that 15 out of 16 CTCs from patient M16 were tiny CTCs. Of the total 42 CTCs, 24 (57.2%) were tiny and 18 (42.8%) were of a normal size.

### 2.6. Correlation between CTC Presence, VDR Status and the Primary Tumor Characteristics

No significant correlation was found between VDR expression on the CTCs and hormone receptor (HR) expression, HER2 status, or triple-negative status of the related primary tumors.

### 2.7. Specific CK Positive Cell Subtypes

**Figure 5:**
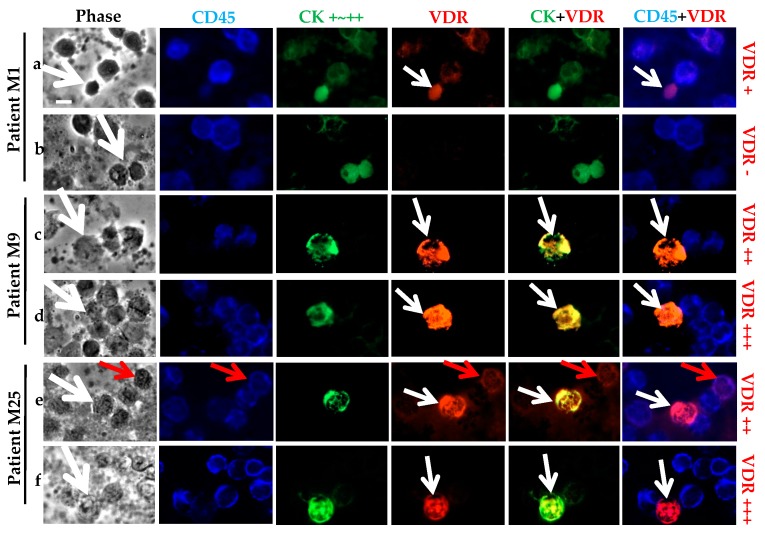
Particular subtypes of CK positive CD45 negative cells. Triple fluorescence labeling of CD45, CK, and VDR was performed on 10^6^ PBMCs from patients, with parallel analysis. Distinct subtypes of CTCs (**with white arrow**) were observed: patient M1 (**a**,**b**) with more than 500 CTCs identified and patients M9 (**c**,**d**) and M25 (**e**,**f**) with superimposed VDR and CK labeling. VDR staining was also seen in PMBCs (**with red arrow**), with superimposed VDR and CD45 labeling. Original magnification, ×40. Scale bar (white bar in the upper left image), 10 μm.

Comparing the morphology of the total 42 CTCs analyzed and within the various CTCs of each individual patient, we observed striking heterogeneities not only in terms of VDR expression, but also with regards to cell size. Besides, we saw very atypical morphologies and fluorescence patterns for cells positive for CK and still negative for CD45. These cells could be CTCs, but we preferred not to include them in our analyses and thus did not report them in Table 2 and Table 3 (Figure 5).

First, the already described numerous CTCs from Patient M1, in which CK and VDR staining is faint and often superposed, can be considered as a distinct CTC subtype compared to the other CTCs (Figure 5, panel a,b). In some other patients, for example patients M9 and M25, the fluorescence labeling of VDR and CK performed a superposition (Figure 5, panel c–f). The phase contrast image of some CTCs showed a specific morphology, with a very faint aspect. As we first suspected them to be fragments or splitting cells, we systematically checked for their morphology and adjusted the exposure time for CK images in order to control and confirm the peripheral staining. We noticed a very high expression of VDR that forced us to decrease the exposure time five-fold (from 1000 ms for the previous pictures of other CTCs to 200 ms in that case) (Figure 5, panel d–f).

## 3. Discussion

VDR has been shown to be expressed in different tissues, as well as in BC cells; however, it has not yet been investigated in BC CTCs from archived specimens. We previously used various human carcinoma cell lines to develop a simple and efficient triple fluorescence technique for CTC receptor analysis, e.g., ER, HER2 [15], *N*-cadherin, and CD133 [26]. We tested the VDR expression in nine BC cell lines and CTCs from 23 archived metastatic BC cases.

The CellSearch^®^ system, which is the only Food and Drug Administration (FDA) approved CTC enumeration method used for clinical purposes, classifies a CTC as a positive event if the nucleated cell is ≥4 µm, pan-CK positive, and CD45 negative [27]. More than a 90% expression of CK7, CK8, CK18, and CK19 was observed in breast carcinomas of all grades, thereby confirming their usefulness for BC identification [28]. Meanwhile, peripheral blood cells (PBCs) such as monocytes (i.e., PBMCs) only express very low mRNA levels of CK (18/19) [29]. Therefore, using a CK antibody combined with a CD45 antibody, a recognized white blood cell (WBC) marker, allows for the characterization of CTCs by CK positive and CD45 negative staining—a technique that has been the most frequently used so far [30]. An additional fluorescence channel is accessible for a user-defined detection of therapy relevant markers. The CellSearch^®^ system (but also other techniques) allows for an analysis of markers such as ER [31], HER2 [15,32,33], epidermal growth factor receptor (EGFR) [34,35], and epithelial-mesenchymal transition (EMT) associated molecules such as *N*-cadherin [26]. Nevertheless, VDR has not yet been evaluated by this technology.

After optimization of the triple fluorescence protocol on BC cell lines, we observed that all nine tested cell lines were VDR positive, as already reported in other studies by Western blot analysis or other techniques [24,36,37,38]. We then characterized the expression heterogeneity among all of the cell lines. Limited publications can be found with regard to the intensity of VDR protein expression in BC cell lines, as most of them focus on mRNA expression [8,39,40,41]. According to our fluorescence analysis, we were able to divide the cell lines into three groups with distinct levels of VDR expression. Standardized identical values of exposure time for optimal pictures of VDR and CK expression were absolutely required for evaluation and comparison (namely 1000 ms for VDR and 2000 ms for CK). As already mentioned in the literature, T47D appeared to be among the high expressing cell lines (although not the highest) and MDA-MB-231 among the low expressing cell lines [36,40]. Besides these differences in the average fluorescence intensity for each cell line, we observed that even within one cell line, individual cells expressed variable VDR levels, thus explaining how mRNA expression cannot always be linked to protein expression. As an example, most MCF-7 cells exhibited a substantial level of VDR fluorescence, but the expression was not identical for all cells. Some cells barely exhibited any fluorescence intensity (2.1%) and others expressed a particularly high intensity (around 4.7%). We speculate that this heterogeneity within one cell line may rely on the cell cycle–related variations of VDR expression or on a wide variety of environmental factors such as cell adhesion and cell density [42,43]. Of note, given the individual heterogeneity of expression within one cell line, we characterized and graded the VDR level by semi-quantifying the average values of different cell lines as the majority of cells showed a consistently low, intermediate, or high intensity.

In order to perform VDR expression analysis on blood samples collected from a consecutive cohort of 23 metastatic BC patients, we first mimicked the CTCs analysis in blood by mixing cancer cell lines with PBMCs from healthy donors. PBMCs are identified as CD45 positive and CK negative, and in our study, only a few of them expressed VDR. This observation is in accordance with the literature stating that VDR expression is controlled by immune signals [44]. It is noteworthy that normal human PBMCs may also express VDR and its target genes [45,46,47]. Using our triple fluorescence method, we detected VDR expression in 42 CTCs, present in 60.8% of our patient cohort with 1 million PBMCs analyzed in each case. Our cohort only consisted of metastatic patients as they have the highest probability of exhibiting CTCs. We first observed very atypical morphologies and fluorescence patterns with cells positive for CK and negative for CD45, and secondly with cells positive for CK and for CD45, which could be CTCs because dual CK and CD45 positive cells are occasionally found in humans [48,49]. Indeed, false-positive CK staining may also occur, as it is possible that antibodies against CK bind to hematopoietic cells through the Fc receptor [50]. We thus decided to exclude both of these groups from further analysis.

All of the studied BC cell lines expressed VDR from low to high levels. Based on the MCF-7 positive controls, we therefore grouped CTCs into two VDR status groups. We could demonstrate that 45.2% (*n* = 19) of the CTCs were VDR positive. In terms of VDR intensity, VDR staining in CTCs was relatively low compared to the high VDR expression observed in some BC cell lines. Optimal vitamin D status has a protective effect against BC development, but epidemiological and early clinical studies are inconsistent. Resistance to vitamin D develops or exists in many BC patients [51]. The anticancer role of vitamin D is mainly mediated by the VDR. Our hypothesis is that VDR may be abnormally (poorly) expressed in BC tissue and/or CTCs. It is known that VDR is lost during carcinogenesis and this may be the reason why tumors become insensitive to vitamin D [25]. Therefore, it would be very helpful to compare the VDR expression of CTCs with that of the corresponding primary tumor. Unfortunately, primary tumor tissue was not available for our cohort. In a previous study [7], Nina Ditch et al. analyzed the relationship between VDR expression in primary tumor tissue and survival in 82 BC patients. Patients with high VDR expression showed significantly better progression-free (PFS) and overall survival (OS) results than patients with moderate/negative VDR expression [7]. In the 13 CTC positive patients of our study, six (46.1%) had at least one VDR positive CTC, with three (23%) patients only having VDR positive CTCs. In contrast, 10 (76.9%) had at least one VDR negative CTC, including seven (53.8%) patients with only VDR negative CTCs. Therefore, we believe that VDR expression may be of clinical significance and that the CTC results need to be correlated to PFS or OS in a larger cohort of patients. Similar conclusions on the role of VDR expression as a prognostic marker have already been addressed in pancreatic cancer and gastric cancer [52,53]. In a large patient population, VDR expression on primary tumor tissue is inversely associated with more aggressive BC including a large tumor size, HR negativity, and triple-negative subtype (*p* < 0.05) [54]. A preclinical study suggested that calcitriol and inecalcitol, an epi-analogue of calcitriol, can inhibit BC cell line growth, especially in cells expressing ER and VDR [55]. This suggests that the VDR-mediated inhibition of ER-positive BC cells may be at least partly affected by the downregulation of ER [56,57,58]. Besides, in contrast to ER-positive cells, treatment with calcitriol was reported to induce the expression of ER in the ER-negative cell line. If confirmed in patients, this ability of calcitriol would have major implications for BC treatment [59]. In order to see whether it could be a potential biomarker, we correlated the VDR expression results observed in CTCs with the related clinicopathological parameters. Most likely due to the small number of patients and only one cytospin analyzed per patient, no significant association was found between VDR expression in CTCs and tumor subtype according to ER, PR, and HER2 status. In further larger studies, it will be essential to correlate VDR expression in CTCs (with at least duplicate cytospins for each patient) with ER, PR, and HER2 status, as well as the VDR itself of the primary tumors. Moreover, repeat analysis of VDR expression on CTCs during the course of disease and after treatments may give very relevant information. Another parameter to consider in parallel will be the serum level of the partially activated 25-hydroxyvitamin D, and consequently 1,25di-hydroxyvitamin D that are expected to be relatively low in BC patients. A large number of studies have concluded that low blood levels of vitamin D are associated with an increased BC incidence and decreased survival in BC [60]. Similar to postmenopausal patients with ER-positive tumors and extremely low serum estrogen levels, it could be speculated that moderate or low levels of 25-hydroxyvitamin D may be sufficient to activate VDR.

The physiopathological significance of the observed CTC size heterogeneity is also a crucial point that may lead to further analyses [61,62]. The Cell Search^®^ system classifies a CTC as a positive event if the nucleated cell is ≥4 μm [27]. We observed 24 (57.2%) CTCs that were “tiny” CTCs (around 5 μm, but higher than 4 μm) and 18 (42.8%) that had a normal size (≥5 μm). Prior publications by us and other research groups have already described and discussed the character of these “tiny” CTCs [15,26,61,63]. There are diverse explanations for “tiny” CTCs: Compared to the size of CTCs in patients with metastases or primary tumors, the size in dormancy candidates is smaller [64,65]. Furthermore, Marrinucci et al. found by DNA disruption analysis and microscopic images that the early apoptosis category of cells contains many CTCs that seem surprisingly small for carcinoma cells, suggesting that these small CTCs are undergoing cell death through apoptosis [66]. Stem cell-like CTCs which are smaller and more aggressive than other CTCs could be another possibility [67]. Similar findings were reported for disseminated tumor cells (DTCs) in bone marrow, where tumor cells with a stem cell-like phenotype were demonstrated [68]. CTCs that are in the process of EMT may be as deformable as WBCs to become more WBC-like and better adapt to the blood flow based on this size issue [69]. These findings likely explain some of the size heterogeneity we observed. However, further studies regarding the exact significance of these “tiny” CTCs in cancer research have not been able to give clear answers yet [70]. Of note, the technical issue of CTC enrichment methods also has to be considered when the methods rely on a size filtration of CTCs from PBMCs [71].

Some specific atypical subtypes of cells were also observed in our study. For example, patient M1’s had more than 500 CTCs in the 1 million PBCMs analyzed, and CK and VDR staining was faint and often superposed. For some CTCs in patients M9 and M25, the fluorescence labeling of CK and VDR performed a superposition with a very high expression of VDR and the phase contrast image of some CTCs shows a specific morphology with a very faint aspect. We speculate that these specific subtypes derive from some stressed, evolving cells that may be going through the apoptosis process: When CTCs are shed from solid tumors, half of these CTCs perish within 2.4 h in vivo [65]. CTCs impaired through apoptotic events exhibit membrane perforation that triggers the leakage of intracellular components, including not only electrolytes or small molecules, but also DNA and chromatin. The technical steps of blood drawing and sample processing can induce the additional stress or degradation of CTCs by various factors. These parameters include the selected purification and analysis techniques, such as temperature shock, fluidic turbulence, shear force, or surface tension. As a result of the various harsh conditions, it is expected that fragmented cells, cellular debris, microparticles, and clump-like aggregates next to “normal” CTCs will be observed [72]. Although we did not include these CTCs in our analysis, they can clearly play a role in the dissemination and metastasis process [26,73,74,75,76].

## 4. Materials and Methods

### 4.1. Cell Culture and Cytospin Preparation

The human adenocarcinoma cell lines MCF-7, T47D, ZR-75-1, and MDA-MB-231, and an endometrial cancer cell line Ishikawa (Heraklio) 02 ER- (Ishikawa ERneg), were obtained from the European Collection of Cell Cultures (ECACC, Salisbury, UK) and the Cama-1 (HTB-21), SK-BR-3 (HTB-30), HCC1937 (CRL-2336), and MDA-MB-468 (HTB-132) cell lines from the American Type Culture Collection (ATCC, Rockville, MD, USA). The HCC 3153 cell line was kindly provided by Adi F. Gazdar (Hamon Center for Therapeutic Oncology Research and Department of Pathology, University of Texas Southwestern Medical Center at Dallas, Dallas, TX, USA). Cryopreservation of cell cultures ranged from passages 1 to 10. Cells were used during up to 20 passages. Cells were grown routinely in Dulbecco’s modified Eagle’s medium (Biochrom, Berlin, Germany), supplemented with 10% FBS (PAA, Pasching, Austria).

For cytospin preparation, trypsinized cells were centrifuged (700 g, 10 min, 4 °C) and resuspended in phosphate-buffered saline (PBS; Biochrom, Berlin, Germany). Then, 1 million cells were spread on each cytospin and centrifuged (45 g, 5 min, room temperature). Cytospins were allowed to dry overnight at room temperature and then stored at −80 °C. They were prepared with either 1 million adenocarcinoma cells or mixed with PBMCs from healthy volunteer donors, as indicated in the legends.

### 4.2. Triple Fluorescence Labeling of CK, VDR, and CD45 with Parallel Phase Analysis

According to the optimized procedure (see Supplementary Material), cytospins were thawed and immediately fixed in 3.7% neutral buffered formalin (Fischar, Saarbrücken, Germany) in PBS for 15 min at room temperature and permeabilized in cold (−20 °C) methanol (Sigma-Aldrich, Steinheim, Germany) for 2 min. After washing in PBS, Ultra V Blocking medium (Thermo Scientific, Fremont, CA, USA) was used for 15 min. This blocking step and all of the following steps were performed in a humidified chamber at room temperature. All antibodies were diluted in Dako Antibody Diluent with Background Reducing Components (Dako, Carpinteria, CA, USA).

As previously described [77], we selected a two-step protocol. Cells were first incubated for 45 min with a monoclonal mouse anti-human VDR antibody (clone 2F4, mouse IgG2a, MCA3543Z, Serotec, Puchheim, Germany) efficiently used in other studies on BC [7,40], washed in PBS, incubated for 30 min with a goat anti-mouse IgG-Fab fragment labeled with Cy3 (Jackson ImmunoResearch, Suffolk, UK), and washed in PBS.

Cells were then incubated for 45 min with a monoclonal rabbit anti-human CD45 antibody (D9M8I, 13917, Cell signaling, Leiden, The Netherlands) and a monoclonal mouse anti-human cytokeratin antibody (Igg1 A45 B/B3, Glycotope, Berlin, Germany), washed in PBS, incubated for 30 min with a goat anti-rabbit IgG labeled with Coumarin-AMCA (Jackson ImmunoResearch) and a goat anti-mouse IgG labeled with DyLight488 (Jackson ImmunoResearch), and washed in PBS.

After drying (30 min, at room temperature), the slides were mounted with Kaiser’s glycerol gelatin (Merck, Darmstadt, Germany) before manual analysis with a computerized fluorescence microscope Axioskop (Carl Zeiss Micro Imaging GmbH, Göttingen, Germany) for phase and fluorescence, with 40× magnification. An AxioCam MR camera and AxioVision software (version AxioVision LE 4.8, Göttingen, Germany) were used to capture, analyze, and save high-resolution images for the three fluorescence channels, considered independently or in combination. Criteria for CK and CD45 positivity were the ring-like appearance (cytoplasm staining in periphery) and we considered that the VDR positivity was always high, average, and low for specific punctuated staining of the nucleus with a low background and no cytoplasmic or peripheral staining. We followed the criteria already described for the identification of CK and CD45 positive CTCs by immunofluorescence [78] and the consensus recommendations for standardized tumor cell detection [79].

Definite threshold values of exposure time for VDR, CD45, and CK fluorochromes were determined, on the basis of the analysis of the cancer cell lines, and were systematically used for the patient analysis described below.

### 4.3. Fluorescence Labeling of VDR and Cytokeratin (CK) with Parallel 4′-6-Diamidino-2-Phenylindole (DAPI) Analysis

Cytospins were thawed, immediately fixed, permeabilized, and blocked as described above.

Cells were incubated as described above, first with a monoclonal mouse anti-human VDR and related secondary anti-mouse IgG-Fab fragment labeled with Cy3, and then with the monoclonal mouse anti-human cytokeratin antibody and the related goat anti-mouse IgG labeled with DyLight488, without the anti-CD45.

After drying (30 min, at room temperature), the slides could be mounted with Vectashield Mounting Medium with DAPI (Vector Laboratories, Burlingame, CA, USA) before manual analysis with a computerized fluorescence microscope Axioskop (Carl Zeiss Micro Imaging GmbH, Göttingen, Germany) with 40× magnification.

### 4.4. Patient Cohort

This analysis was performed at the Department of Obstetrics and Gynecology, Ludwig Maximilian University (Munich, Germany). Twenty-nine metastatic BC patients were recruited between May 2010 to July 2012; with six exclusions (patient M5 with a cancer of unknown primary origin (CUP) syndrome and patient M3, M5, M14, M21, and M23 with an insufficient blood sample). Table 2 describes the characteristics of the final cohort of 23 patients and their primary tumors. Written consent forms were collected from the patients using protocols approved by the institutional ethics committee (approval number 148-12; 12.05.2012, Ethikkommission bei der Ludwig-Maximilians-Universität, Munich). The phenotype of the primary tumor was routinely assessed at the time of diagnosis by immunohistochemical staining and potentially by FISH, Fluorescence In Situ Hybridization (for most HER2 ++ patients) in the original Department of Pathology and collected from the patient files. ERα status was classified by an evaluation of the percentage of tumor-stained cells and staining intensity, allowing for an assessment of an Immunoreactive Score (% score × intensity score). HER2-negative assessment include 0 or + staining and ++ staining with FISH-negative amplification and HER2-positive assessments include ++ with FISH amplification or +++ staining. The ERα and HER2 status were indicated in Table 2, if available. Because the HER2 status of the primary tumor of patient P7 was not determined in 1999, at the time of diagnosis, we considered the HER2-negative status of the local recurrence assessed in 2009, with ERα positivity and PR negativity recorded for the primary tumor.

### 4.5. Blood Sampling, Ficoll and Cytospin Preparation

Fifteen milliliters of blood from each patient was collected by needle aspiration and placed in EDTA tubes. The blood was processed by a modified Ficoll protocol, with Ficoll-Hypaque (Pharmacia, Erlangten, Germany) density gradient centrifugation (density 1.007 g/mol) at 900× *g* for 30 min [80]; the mononucleated cells or PBMCs, were counted and centrifuged (700 g, 10 min at 4 °C), and then 1 million cells were spread out on each cytospin and centrifuged (45× *g*, 5 min, room temperature), before being processed as described above.

### 4.6. CTC Analysis by Triple Fluorescence

The triple fluorescence labeling of VDR, CK, and CD45 with parallel phase analysis was performed on the cytospins prepared from patient blood, as described above. The preparation of BC cell lines MCF-7 was mixed with PBMCs from healthy donors, in which MCF-7 cells served as a positive control for CK and VDR stainings and as a negative control for CD45; Mixed PBMCs served as a positive control for CD45 staining and as a negative control for CK. In each batch of patient samples we analyzed, one MCF-7 mixed with the PBMCs control slide was systematically performed. The screening of CTCs through CK staining was performed using a 20× magnification to get an optimal sensitivity, and the VDR and CD45 expressions were then assessed using a 40× magnification. For each patient, one cytospin was analyzed (1 million cells per patient) and each slide was evaluated by two independent investigators, and three in doubtful cases (X. Zhang, S. Sixou and U. Jeschke). For one patient (4.3%), the evaluation of the two observers differed for either the CTC detection or VDR positivity. These cases were re-evaluated by the three observers together. After the re-evaluation, the observers came to the same result. The concordance before the re-evaluation was 95.7%. The analysis was always performed within 72 h after the labeling procedure. Each observed CTC was recorded with at least one picture for each channel of analysis.

### 4.7. Statistical Analysis

A Fisher exact probability test was used to evaluate the relationship between the receptor status of the primary tumor and the VDR expression of the CTC for the 14 CTC-positive patients. *p* < 0.05 was considered statistically significant.

## 5. Conclusions

In this study, we demonstrated the evaluation of VDR expression from BC cell models to CTCs of metastatic BC patients. CTCs are a promising marker, providing important predictive and prognostic information in both early and metastatic BC. To the best of our knowledge, this work is the first study about VDR status on CTCs from BC patients. This preliminary study gives a direction for further VDR exploration, suggesting that prospective larger studies should be performed in the future. This will help elucidate VDR profiling in BC, including a parallel analysis of vitamin D and its receptor in CTCs and the corresponding primary tumors. Eventually, VDR may serve as a new prognostic biomarker in BC and a promising target for innovative BC therapies.

## Figures and Tables

**Table 1:** ER, progesterone receptor (PR), HER2, and VDR expression levels of the nine BC and one endometrial cell lines.

Cell Line	ER	PR	HER2	VDR Level
MDA-MB-231	−	−	−	high
MCF-7	+	+	−	high
Cama-1	+	+	−	high
T47D	+	+	−	intermediate
HCC1937	−	−	−	intermediate
ZR75	+	−	−	intermediate
SK-Br-3	−	−	+	intermediate
Ishikawa ERneg	−	−	−	intermediate
MDA-MB-468	−	−	−	low
HCC3153	−	−	−	low

BC: breast cancer; ER: estrogen receptor; PR: progesterone receptor; VDR: vitamin D receptor; −: negative; +: positive.

**Table 2:** Patient characteristics and CTC presence.

Patient	Age *	Primary Tumor Status						Primary Diagnosis ~Metastasis (years) **	Primary Diagnosis ~CTC Analysis (years) ***	CTCs
		TNM		Grade	ERα	PR	HER2			
M1 ^CTC^	56	pT2, pN2, M0		G2	+	+	−	5 and 10	14	>500
M2	62	cT4, N3, M1		G3	−	−	+	0	5	-
M4	69	cT3, pNx, pM1		G3	−	−	+	0 and 1	5	-
M6 ^CTC^	49	ypTis, pN1, M0		G3	−	−	+	2	7	1
M7 ^CTC^	75	pT2, pN1, M0		n.d.	+	−	− ****	7	16	3
M8 ^CTC^	53	pT1b, pN0, M0		G2	+	+	−	8	16	5
M9 ^CTC^	63	pT1c, pN0, M0		G2	+	+	−	8	16	1
M10 ^CTC^	69	Left	pT2, pN0, M0	G2	+	−	+	5 and 6	10	1
		Right	pT1c, pNx, M0	G2	+	−	+			
M11 ^CTC^	66	ypT2, pN3a, M0		G3	+	+	−	2 and 3	9	1
M12	63	ypT2, pN2, M0		G3	−	-	+	3	11	-
M13 ^CTC^	77	pT2, pN1, M0		G2	+	+	−	2 and 6	11	1
M15	82	cT3, cN1, cM1		G2	+	+	+	0	5	-
M16 ^CTC^	69	pT1c, pN0, M0		n.d.	+	+	−	10 and 12	16	16
M17 ^CTC^	64	pT1c, pN0, M0		n.d.	+	+	+	6	13	1
M18 ^CTC^	70	pT4b, pN1, pM1		G2	+	+	−	0	4	2
M19	69	pT2, N0, M0		G2	−	-	+	0	4	-
M20	61	pT3, pN3, M1		n.d.	+	−	−	0 and 1	5	-
M22	53	pT2, pN0, M0		G3	−	−	−	4	8	-
M25 ^CTC^	73	Left	pT1c, pN0, M0	G1	+	+	−	6	10	8
		Right	pT2, pN0, M0	G2	−	−	−			
M26	46	ypT0, pN1a, M0		G2	+	+	−	1	5	-
M27 ^CTC^	59	Left	pT2, pN1, M1	G2	+	+	−	3	7	1
		Right	pTis, pNx, M1	n.d.	n.d.	n.d.	n.d.			
M28 ^CTC^	79	ypT3, pN1a, M0		G2	+	+	−	9	13	1
M29	67	pT1c, pN0, M0		G3	−	−	−	0, 2 and 5	15	-

n.d. indicates not determined; * indicates at time of CTC analysis; ** indicates time between primary diagnosis and metastasis (years); *** indicates time between primary diagnosis and CTC analysis (years); **** indicates no information for the primary tumor, but HER2-negative recurrence 10 years later (ERα-positive and PR-negative as the primary tumor); ^CTC^ indicates CTC positive patients. CTCs: circulating tumor cells, −: negative; +: positive; -: absence.

**Table 3:** Characteristics of the CTCs found in 14 patients.

Patients with CTCs	CTCs (*n* = 42 *)				Total
	Tiny CK pos		Normal CK pos		
	VDR		VDR		
	Neg	Pos +~++	Neg	Pos +~++	
M 1					>500
M 6			1		1
M 7	1		1	1	3
M 8	1		4		5
M 9	1				1
M 10	1				1
M 11				1	1
M 13	1				1
M 16	3	12	1		16
M 17				1	1
M 18	2				2
M 25	1		4	3	8
M 27				1	1
M 28	1				1
Total	12	12	11	7	42 *
% (*n* = 13)	28.6	28.6	26.2	16.6	100 *

* Indicates without taking into account the CTCs from patient M1. CK: cytokeratin, Pos: positive; Neg: negative.

## Supplementary Materials

Supplementary materials can be found at www.mdpi.com/1422-0067/18/6/1318/s1.

## Abbreviations

BC	Breast cancer
CTC	Circulating tumor cell
CK	Cytokeratin
EMT	Epithelial–mesenchymal transition
ER	Estrogen receptor
FDA	Food and drug administration
FISH	Fluorescence in situ hybridization
HER2	Human epidermal growth factor receptor 2
HR	Hormone receptor
NR	Nuclear receptor
OS	Overall survival
PBC	Peripheral blood cell
PBMC	Peripheral blood mononuclear cell
PFS	Progression free survival
PR	Progesterone receptor
RAR	Retinoic acid receptor
RXR	Retinoid X receptor
THR	Thyroid hormone receptor
VDRE	Vitamin D response element
VDR	Vitamin D receptor
WBC	White blood cell
