# Supplementary material for: Fluorescence Analysis of Vitamin D Receptor Status of Circulating Tumor Cells (CTCS) in Breast Cancer: From Cell Models to Metastatic Patients
Source: Int J Mol Sci. 2017 Jun 20;18(6):1318. doi: 10.3390/ijms18061318 (PMC5486139; doi:10.3390/ijms18061318)
Supplement: Supplementary file 1 [file ijms-18-01318-s001.zip › ijms-199212-supplementary.docx]

Supplementary Materials

Fluorescence Analysis of Vitamin D Receptor Status of Circulating Tumor Cells (CTCS) in Breast Cancer: from Cell Models to Metastatic Patients

Xi Zhang ^1^, Simone Hofmann ^1^, Brigitte Rack ^1^, Nadia Harbeck ^1^, Udo Jeschke ^1^ and Sophie Sixou ^1, 2^*

Optimization of the Triple Fluorescence Labeling of CK, VDR, and CD45

Starting from the fluorescence staining procedure of CK, ERα and HER2 developed in our laboratory [15], various parameters have been optimized. We used MCF-7 and T47D BC cell lines mixed with PBCs. We used the following cell fixation and permeabilization procedure: fixation in 3.7% neutral buffered formalin in PBS for 15 min at room temperature and permeabilization in cold (−20 °C) methanol for 2 min. Then, we tested primary antibodies: against VDR, a monoclonal mouse antibody (klon 2F4, mouse IgG2a, Serotec, MCA3543Z) with the second antibody being a goat anti-mouse IgG-Fab fragment labeled with Cy3 (Jackson ImmunoResearch); against CD45, a polyclonal rabbit antibody (ab10558, Abcam) and a monoclonal rabbit antibody (D9M8I, 13917, Cell signaling) with the second antibody being a goat anti-rabbit IgG labeled with Coumarin-AMCA (Jackson ImmunoResearch); against cytokeratin, two monoclonal mouse Antibodies ((PCK-26) (ab6401), Abcam) and (Igg1 A45 B/B3, Glycotope)) with the second antibody being a goat anti-mouse IgG labeled with DyLight488 (Jackson ImmunoResearch).

Both antibodies designed against cytokeratin were purified monoclonal pan-cytokeratin antibodies, which specifically detect the epithelial cytoskeleton components CK8, CK18 and CK19. In parallel, we tested different antibody dilutions (from 1:50 to 1:1000) with Dako Antibody Diluent with Background Reducing Components (Dako, Carpinteria, CA). Incubation times for primary and second antibodies varied from 30 to 90 min and overnight. Then we tried different blocking reagents, Ultra V Blocking medium (Thermo Scientific, Fremont, CA) and Power Block (HK085-5K: Biogenex, Fremont, CA). Once the labeling procedure was set for VDR, CD45 and CK, we checked that cytospins frozen for at least 2 years could be used with no alteration of the staining and that, once stained, fluorescence of cytospins stored at + 4 °C could be visualized, with no significant loss of fluorescence, during at least 96 h (data not shown). Analyses presented here were nonetheless always performed within 48 hours after the labeling.

© 2017 by the authors. Submitted for possible open access publication under the
terms and conditions of the Creative Commons Attribution (CC BY) license (http://creativecommons.org/licenses/by/4.0/).
